# Select Dissertations on Several Subjects of Medical Science

**Published:** 1823-03-01

**Authors:** 


*Select Dissertations on several Subjects of Medical Science.* By Sir Gilbert Blane, Bart. F.R.S. Physician to the King, &c. Now first collected, with Alterations and Additions; together with several new and original Papers. Octavo, pp. 398. London, Underwoods, Nov. 1822.

These Dissertations are twelve in number, of which eight are republications, (some of them with additions and alterations,) and four are now published for the first time. On calculating by the pages, the original articles occupy about 100, and the republications 286 pages. Every man has an undoubted right to collect together the fragments of his literary labours, and publish them as a whole; but we question the policy, in such cases, of interweaving new matter. Those who are already in possession of the original essays may consider it hard that they must re-purchase eight essays out of twelve, or else be deprived of the other four. We think the better plan, both for the author and for the public, on such occasions, is to publish the old and the new essays separately, and thus suit the circumstances of all classes, This would have been peculiarly desirable in the present case, because

Sir Gilbert's character for science and erudition has rendered all his published essays very extensively known throughout the profession, and few will like to be deprived of the pleasure and instruction derivable from any thing new from the same pen. It is therefore, we repeat it, a great pity that he has imposed a penalty on the purchase of the new matter contained in the volume before us.

The first dissertation, on the comparative health of the navy from 1779 to 1814, was published, or the greater part of it, in the sixth volume of the Medico-Chirurgical Transactions. The second dissertation is a sequel or appendix to the former, being a memoir on the medical service of the fleet in the West Indies in the year 1782. Here we see the workings of human nature. As we advance in life we look back and cling to those transactions in which we took a part in youth. The memorable 12th of April, 1782, was an epoch which might well make the heart of an Englishman (especially if attached to the naval service) glow with pride, on recollection of that famous day. We are not therefore surprized to find Sir Gilbert, who stood the brunt of Count de Grasse's thunderbolts on the quarter deck of the Formidable,* recal and recapitulate scenes and events that must have made a very vivid impression on the youthful mind. So far from censuring our veteran author for introducing some circumstances apparently calculated to gratify personal vanity, we are sorry he has not laid before us more of these auto-biographical sketches, which must be read with interest by all members of that profession to which Sir Gilbert Blane has always been an honour and an ornament.

Although the dissertation in question contains many observations that are very worthy of being read and reflected on by officers, civil, military, or medical, who are in any way concerned in the preservation of the lives of seamen or soldiers, yet in these "piping times of peace," we dare hardly dwell upon them, to the exclusion of matters that come home more immediately to the interests and occupa-

---

* By the way we have no hesitation in questioning the propriety of Lord Rodney's conduct in permitting the physician of the fleet to station himself on the quarter-deck of the Formidable, during the action of the 12th April. It surely is defeating the intentions of Government and humanity to permit a medical officer to place himself in the same predicament of danger as a common sailor or marine, without any earthly advantage to be derived from the circumstance. Every other life can be easily replaced during action, except that of the medical officer, and no consideration should induce the latter to ask, or the commander to grant permission for this unnecessary exposure.—*Ed.*

tions of the great bulk of our readers. The following little anecdote we shall extract as a specimen of French vivacity under circumstances little calculated to give origin to sallies of wit.

"It is further observed, that this nation bears adversity with more equanimity than the English. An eminent example of this occurred to my own observation in the case of the Comte de Grasse, commander-in-chief of the French fleet, who was taken prisoner in the Ville de Paris. When he was conveyed on board of the Formidable the morning after the battle, the first conversation was carried on with Lord Rodney, through Sir Charles Douglas; for our Admiral had never learned to speak French; but Sir Charles being much engaged in the duties of the fleet, beckoned to me to replace him as interpreter, introducing me to him in the following facetious manner:—'*Permittez moi, mon Géneral, de vous présenter notre medecin en chef, qui est presque assez habile pour faire revivre les morts;*' to which the Comte, humouring the *plaisanterie*, answered, '*Et peut-être pour faire mourir les vivans.*' It fell to my lot chiefly to entertain him during the rest of the day, and his conversation partook of the like affability and good humour." 80.

There are some physiological observations in the succeeding page, where we fully agree in sentiment with the illustrious author. He observes that, time immemorial, it has been the established practice in the British Navy to allow no other refreshment, during battle, than plain water. Long experience has confirmed the propriety of this practice—not only in battles, but in all violent exertions of the muscular, and excitement of the intellectual systems. In military combats both these are combined, and stimulants, whether vinous or spirituous, are injurious, as quickly exhausting the energy of the system, already too rapidly tending to that point. Even food is injurious; for the whole of the vital powers are now concentrated in the nervous and muscular systems, and the digestive organs are not in a state to perform their functions—or if they are excited to action under the circumstances in question, there is a correspondent subduction of energy from the sensorial and muscular apparatus, where they are most required in the conflicts of war. Tea or coffee are the only refreshments that are at all desirable in the heat of battle or immediately after its termination. In the revolutionary war brandy was very generally exhibited to the French soldiery before coming to the charge. This, united with the natural impetuosity of the people, produced a super-human excitement amounting almost to a temporary madness—but this state could not last long, and if cool intrepidity resisted the first shock of this physical fulmination, its triumph was certain. Many were the illustrations of this in the late contests between France and England!

The great improvement in the health of fleets and armies during the last thirty or forty years, but particularly during the last fifteen or twenty years, is truly surprising; and forms a theme of gratulation for every philanthropist. It is not less important also to the statesman; for in all former ages, there was no more common cause of failure in great armaments and expeditions than disease. Sir Richard Hawkins, an eminent commander and navigator in the reign of Queen Elizabeth, states that, in the course of twenty years, he had known of *ten thousand men* having perished by scurvy alone! This will appear prodigious, when we recollect that the navy was not then one-twentieth part of what it was at the conclusion of the late war! The histories of Hosier, Vernon, and Anson will afford melancholy examples of the contrast to our present state of health and security on expeditions. The fatal Walcheren enterprize can hardly be brought up in opposition, because our troops there suffered from the local endemic, and not from the effects of sea voyages or sea diseases.

"The late revolutionary war may be said to form a contrast with all preceding wars in point of health, and its unexampled glories are in no small degree imputable to this. And it is to be hoped that the methods of securing this invaluable blessing are now so rooted in the practical habits, experience, and convictions of naval officers of all descriptions; that, those scenes of misery and disaster which have been quoted from history, and which rend the heart in the narration, can never recur, should the nation ever again be involved in war; which in the common course of human affairs, can hardly be doubted." 86.

The third dissertation is on the Walcheren expedition in 1809* Sir Gilbert was sent there by Government in the autumn of the year above-mentioned, and remained in Walcheren and Beveland a fortnight. We were present in this fatal expedition from beginning to end; and we can confirm the justice of Sir Gilbert's remarks as far as they extend. The shortness of his sojourn there prevented him from making a great many more observations, that would have occurred to a mind like his in respect to the etiology, pathology, and treatment of so fatal an endemic.

The fourth dissertation "on the comparative prevalence and mortality of different diseases in London," has been be-

---

* Published in the Medico-Chirurgical Transactions for 1812.

fore the public since the year 1813,* and is acknowledged to be a very able and interesting document. Our author states that in this republication "many new facts and illustrations are added." We have not time to collate the two editions in order to remark on these last; but the paper as it now stands is certainly very creditable to the learning and talents of this veteran physician.

The fifth dissertation is new, and forms a kind of appendix or sequel to the fourth; but it is almost exclusively politico-economical in its nature, and therefore it affords us few materials for analysis in a work that keeps so closely to matters purely medical, and more especially practical. By a table appended to this dissertation, and constructed by that accurate calculator Mr. Finlaison, late of the Admiralty, it would appear that within little more than one hundred years the value, or, in other words, the mean duration of human life has increased about ten years. Thus the mean duration of life, at five years old, was calculated to be 41 years, in the year 1693; whereas, in the year 1789, it was calculated at 51 years. At the age of ten, it was enhanced fron 38 to 48—at twenty, from 31 to 41—at thirty, from 27 to 36—at forty, from 22 to 29—at fifty, from 17 to 22—at sixty, from 12 to 15—at seventy, from 7 to 10.

If no fallacy have crept into the foregoing calculations, (and Sir Gilbert asserts that the conclusions are demonstrations themselves,) they may well excite our surprise and satisfaction.

"The causes," says our author, "appear chiefly referable to the more ample supply of food, clothing, and fuel; better habitations; improved habits of cleanliness and ventilation in persons and houses; greater sobriety, and improved medical practice. Whether these causes operate with a relative degree of effect corresponding to the order in which they here stand, or any other order, must be matter of opinion; but if health and long life are to be admitted as the surest criterions and constituent elements of human happiness, it would appear that we have much reason for self-congratulation in having had our lot cast in this age and country." 181.

The 6th dissertation is a re-publication from the second and third volumes of the transactions of a Society for the Improvement of Medical and Chirurgical Knowledge. The paper is in two parts, the *first* on the effect of large doses of the carbonates of potash in gravel, with remarks on the administration of opium: the *second*, on the use of pure alkalies and lime water in disorders of the stomach, bladder, and

* See Med. Chir. Transactions, vol. iv.

skin. To these dissertations Sir Gilbert has appended notes, and introduced remarks corresponding with our present state of knowledge on the subjects abovementioned.

An important addition to alkalies in calculous complaints, in Sir Gilbert's experience, is opium—a medicine that not only allayed pain and irritation, but "contributed materially to expedite and complete the cure." He was induced to adopt this practice from finding that a medicine sold as a secret, "evidently consisting of alkali combined with opium, had, in some cases of gravel, been more effectual than the alkali directed by himself without this addition."

> "I have, therefore, for several years been in the habit of adding from seven to fifteen drops of vinum opii to each of the doses of the alkali, and am fully satisfied, that it not only prevents the distress arising from irritation, and facilitates the discharge of calculi, by relaxing the spasms of the ureters, but that it renders the cure more expeditious, more certain, and more permanent. In those constitutions which do not bear opium, hemlock has been found a useful substitute. I recollect hearing Dr. Black, in his Lectures on Chemistry, which I attended in the year 1770, in the University of Edinburgh, mention that hemlock was a remedy in gravel, but whether in his own experience or not, my memory does not serve me. Dr. Prout, in an ingenious and elaborate work on urinary concretions published last year, (1821,) says that hyosciamus is eminently useful, particularly in those cases of concretions in which lithate of ammonia prevails." 185.

Our author's experience corroborates that of others respecting the utility of opium in irritable and painful ulcers—especially in phagedenic buboes, and some cases of sloughing chancres. He justly remarks that as all healing processes are ultimately and essentially the work of Nature, so the means of art consist merely in enabling Nature to perform these processes, or remove those obstacles that impede her operations. Our chief obstacle is *irritation*. "Upon this principle it can as easily be conceived how the morbid action generating gravel may be increased by the irritation of the gravel itself, as that a sanious discharge should be kept up and increased by its own acrimony." We have little doubt indeed that the action of most, if not all lithontriptics, is more functional than chemical—that they operate more through the medium of the digestive organs than by chemically affecting the products of renal secretion. That chemical experiments out of the body are very fallacious, Sir Gilbert relates the following fact:—

> "A gentleman, subject to frequent fits of gravel, was in the habit of making experiments on the small concretions which he passed. He found that soda dissolved these, but that potash did not; nevertheless, he experienced sensible relief, and even temporary cure, from the internal use of the latter, but no benefit from the former."* 188.

Our author prefers the potash to the soda in calculous complaints in general.

Among many judicious remarks on opium we find it stated by our author that "he has not seen it manifest any of its peculiar properties, whether local or general; that is, any narcotic, anodyne, anti-spasmodic, or exhilarating effects, except when brought into contact with some portion of the alimentary canal." Surely Sir Gilbert cannot be serious here. We have seen both solid and liquid opium ease pain when externally applied as often as we have hairs on our head; and we fancy there are few of our readers who have not seen the same. Has not every one seen the momentous effects of strong solution of opium, hyoscyamus, conium, &c. when applied in fomentation, where the hot water alone would have no adequate effect? We refer also to Ward on opiate frictions for further illustration. For our author's observations on the use of alkalies and lime water in irritable bladder, indigestion, and cutaneous affections, we must refer to the work itself, or to the volumes of Transactions already quoted.

The 7th dissertation is on infection or contagion; and though an original article in the volume, it would be unreasonable to expect much originality on a subject which has been completely exhausted—we are sorry to say without fixed conclusions being come to on many important points. As this section is almost entirely ratiocinative and historical, we could give no fair account of it, and must therefore pass it by. On the great majority of doctrinal points we agree with the learned author—and for the rest we leave them to future tribunals for determination.

The 8th dissertation is on muscular motion, delivered as the Croonian lecture thirty-four years ago. It is too well known to require notice in this place. There are many notes, however, appended, and considerable additions made to the original lecture.

The 9th dissertation is on the yellow fever; but as "the greater part of this dissertation will be found in the first edition of Medical Logic," and as the subject was taken into consideration rather fully in the sixth number of our

* A remarkable instance of the inefficacy of soda, though given in large quantity, is related in Mr. Home's Observations on Mr. Brande's Paper on the Structure of Calculi, inserted in the Philosophical Transactions for 1808."

*Quarterly Series* for October 1819, we do not deem it necessary to renew the discussion here. We certainly were not a little surprised to see Sir Gilbert offer as a reason for omitting the greater part of the yellow fever discussion in the *second* edition of his Medical Logic, a belief that the question was now decided, and that all parties had come to the creed of contagion! The parties, we apprehend, have rather too much of "opposition stuff" in their compositions to be thus so suddenly blended and neutralized, even by the Logic of the worthy Baronet. This he now finds, and like a veteran warrior he returns to the charge, acknowledging that he had calculated without his host in supposing the enemy vanquished.* The following passage may perhaps shew that Sir Gilbert is not very far, after all, from enrolling himself with the small band of "contingent contagionists," among whom we have always numbered ourselves.

"From my own observation in the islands from the year 1780 to 1783, I had not much opportunity of seeing it in its worst forms. The navy as well as the army, and civil population, were more than ordinarily exempt from it in these years, insomuch, *that I saw more cases of what is popularly called the Yellow Fever, which were not of an infectious nature, than of those which were so.* I saw enough, however, in the hospital at Barbadoes, and in the ships and hospital at Jamaica, to convince me of its contagious nature in certain circumstances; and from the best consideration I have since been able to give this subject, I remain persuaded that whenever it is so aggravated as to appear in an epidemic and pestilential form it is truly contagious." 285.

We shall not make more than one or two remarks on this subject. Sir Gilbert allows that he had very few opportunities of seeing the disease in its worst forms, and that in the *majority* of cases which came under his notice the disease was *not* contagious. Now in the majority of cases the fever must have arisen from some cause or causes in the earth or air, for contagion he puts out of the question; and if so, we ask Sir Gilbert, and every unprejudiced man, why it should not arise from similar causes on a large as well as on a small scale? Why, we ask, is the fever *necessarily* contagious because a thousand happen to be stricken with it in a day, in various and distant parts of a city or district, instead of a hundred or fifty, or ten? The logic by which we come to such a conclusion may be medical logic, but assuredly it is bad logic.

* "I was then (in the second edition) under the belief that the opinion of non-contagion had been nearly eradicated, but finding this not to be the case, I have deemed it my indispensible duty, &c." P. 286.

In the absence of demonstrable proof, we should be more inclined to come to the opposite conclusion, namely, that where there are only a *few* cases of the fever it might arise from contagion in the form of fomites; but where a whole people are suddenly assailed, that the cause was a general one conveyed through the air, or springing from the soil. That, in the latter case, (epidemic) an additional source of the disease is occasionally formed by filth, crowding, and want of ventilation, in the form of contagion, we readily admit, and firmly believe; but that the fever should *necessarily* be contagious because it is epidemic, is a creed to which we cannot subscribe. It is like too many creeds in this world—the offspring of credulity or prejudice. We are sorry to observe that even a Sir Gilbert Blane is not free from something approaching illiberality, when his darling doctrine of contagion is in question. Thus, in alluding to a very excellent article on yellow fever in the ninth volume of the Medico-Chirurgical Transactions, our author makes use of the following language:—

> "The *shallow and perverted reasoning of this and some other authors*, would not have claimed notice, but as what they give to the world may prove mischievous, by some inexperienced or weak practitioner, applying what is advanced by them, to the pestilential epidemic fever, it becomes the peculiar duty of one, who has been forty years in the medical service of the State, to counteract the baneful impression it may make; and as no example more apt could be selected to illustrate the necessity of verbal precision and discrimination in medical reasoning." 290.

Of these forty years spent in the service of the State, we apprehend that little less than thirty-six were passed in London. Our author was about three years in the West Indies, at a period of unusual salubrity, and where he had, on his own shewing, very little experience of yellow fever. We do not suppose that Sir Gilbert will quote the time during which he was a medical commissioner at Somerset Place, as particularly fraught with opportunity of observing yellow fever, and therefore we cannot but regard this *forty years experience* as a knock-down argument very much beneath the dignity of science, and very unfortunately chosen, when the extremely limited sphere of observation of our author is made known. In fine, we cannot help making the remark that, in our humble opinion, Sir Gilbert's knowledge of yellow fever is very scanty, and his opinions very prejudiced.

If our author complains of this language, let him compare it with his own in the extract just quoted. We appeal to himself which of the two is the more severe. We are sorry to be obliged to express ourselves with any thing like warmth upon this occasion; but we too have a duty to perform, as well as Sir Gilbert, and the public will probably give us credit for being equally as anxious as he is to establish truth, and "counteract baleful impressions."

Sir Gilbert cannot accuse us of being exclusive non-contagionists. We have, ever since we were capable of forming a judgment on the subject, considered the exclusive contagionists and non-contagionists as equally in error. Diseases and their causes are not so simple and uniform as either party represent them. Fevers will arise from *general* causes, and they will be propagated afterwards by *local* ones:—in other words, fevers, endemic or epidemic in their origin, will become contagious in their course—and thus keep up the ball of contention among the *ultras* of both parties. It is these last, by the way, who chiefly come before the public in brawling controversies. We are convinced that the great body of practitioners, who write little on the subject, are of the moderate way of thinking, and view the question in pretty much the same light as we do.

As our author admits that his practical knowledge of yellow fever is very limited, he makes but two therapeutical remarks, and these we shall give in his own words.

"The first he will mention is, that he has some reason to believe that a course of mercury, previous to entering the Carribean station, has the effect of removing, or at least of lessening the susceptibility to the yellow fever. The chief fact upon which he founds this, is what occurred in one of the ships of war which arrived in the West Indies towards the end of the American war, in which a great proportion of the crew had been under a course of mercury on the passage; and it was remarked, that after these men arrived and were exposed to the same cause of sickness as the others, they were the only persons who escaped the fever of the climate. It would appear, that the mercury had reduced their constitution to the standard of the coloured and Creole inhabitants, and of the seasoned Europeans. The Author has no experience of his own on this subject, and leaves it to others to pay such regard to it as their own judgment may dictate.

"The other remark he has to make, respects certain violent methods of practice which have been followed, even at a very late period, in the tropical fevers. He alludes particularly to the severe depletory remedies of bleeding and purging, especially the former, which have been employed without that discriminating judgment which is indispensable in all sound practice. His attention has been very strongly drawn to this subject, by lately reading the detail of a most afflicting case of a young naval commander, on the West India station. He was seized a few weeks after his arrival with a continued fever, the symptoms of which were by no means violent or alarming, nor was he of an athletic frame. The practice followed was that of bleeding every day, from one to two pounds for the first four days of his illness, and two pounds two days before his death, which happened on the sixth day. The whole quantity of blood abstracted amounted to a gallon. He was also repeatedly and severely purged with salts and calomel. This is the revival of a practice, which had been tried and abandoned on account of its want of success, at the beginning of the great epidemic in 1793. The author does not mean here to explode depletory remedies in all cases; on the contrary, he is well assured, from his own experience, and that of others, that a single bleeding in the first day, or at farthest, the second day of attack, may be highly useful in the case of strong plethoric subjects newly arrived from Europe. It is to be lamented, that the most cautious and judicious employment of remedies have not made any sensible difference in the general rate of mortality in this most malignant disorder; but it is truly humiliating to reflect, that it should be increased by rash and undistinguishing practitioners. Will the junior members of the Medical Service of the Navy forgive a veteran in that department, who has zealously devoted to it a large portion of a long life, for warning and imploring them not to give lightly into such outrageous and indiscriminate practices, as they regard the sufferings and value the lives of their fellow men who are entrusted to their care: not to mention what is above all price in the estimation of every good man—peace of mind, and a clear conscience." 333.

To a certain extent we agree with Sir Gilbert in condemning the indiscriminate, and still more the *reiterated and protracted* bleedings which have been resorted to in yellow fever, as if it was a mere topical *inflammation*, for which the lancet must *continue* to be used as long as the symptoms remain. We are advocates for liberal depletiou at the very onset of the disease, where the reaction is violent; but we deprecate a perseverance in sanguineous evacuations beyond the first or second day.

The 10th dissertation is on vaccination, published in the Medico-Chirurgical Transactions for the year 1819. It has also been re-printed, with some additions, in the year 1820, by the desire and at the expense of the venerable discoverer of this invaluable preventive of variola. It would be needless for us to farther notice a paper so widely circulated and so favourably known among the profession.

The 11th dissertation contains the narration of a hurricane in the West Indies. This is not noticed as a re-publication by Sir Gilbert; but we suspect that it has before appeared in print; as it is in the form of a letter from Sir Gilbert to Dr. William Hunter, dated December 22d, 1780. Probably it was only published in some of the ephemerals of the day, and is now rescued from oblivion by its parent.* These

* This is very natural. As age advances, we look back with mellowed awful *extravaganzas* of Nature have but little connexion with medical science; and with a journal of practical medicine still less. The following extract contains all the strictly medical part of this dissertation.

"The influence of this general tumult of nature upon the health of man is none of the least curious of its effects. I have made much enquiry upon this head, not only at the medical Superintendants of the Naval and Military Hospitals, and the physicians of the place, but at private persons; and I find, that so far from its having been productive of sickness, there has been less of it since, and even that most of those who laboured under disease at the time benefited by it, except the very old and delicate, who suffered from mechanical violence, or the subsequent want of shelter. This is a fact so paradoxical, that if I had not a concurrence of testimony, and in some degree my own observation, I could neither credit, nor would venture to relate it. It had a visibly good effect on the diseases of the country; fevers, fluxes, and chronic diarrhœas the consequence of dysenteries, were also cured by it. But the diseases upon which it operated most visibly and sensibly were pulmonic complaints. Some cases, supposed to be beginning consumptions, and even the acute state of pleurisy, were cured by it. In the more advanced and incurable state, the hectic fever was in a great measure removed, and a temporary alleviation at least procured. A delicate lady, of my acquaintance, was ill of a pleurisy at the time, and passed more than ten hours in the open air, sitting generally in a plash of water, from the rain that fell; she had no more of her complaint, nor any return of it; and I saw her a few weeks after, in better looks and general health than she had enjoyed for a great while before. It was a general observation, that people had remarkably keen appetites for several days after, and a number of those whom I knew, formerly thin and sallow, looked fresh and plump a few weeks after, though the unhealthy rainy season was then hardly over." 370.

Sir Gilbert justly suspects that the agitation of mind, consequent on such a tremendous conflict of the elements, must have played its part, not only in averting the effects naturally to be expected from exposure to the cold and rain, but in removing many physical ailments previously existing. "Neither is it ridiculous to suppose that the purity and coolness of the air would have a happy effect on the animal frame,

---

pleasure on all the prominent events of that period of our existence when "life itself was new;" and therefore it is no wonder that we should feel a secret gratification in recounting our dangers, recalling to mind our associates, and reviewing our personal histories.

Should auld adventures be forgot,

And never brought to mind;—

Should auld acquaintance be forgot,

And days of Lang Syne!

especially as the diseases of the lungs were most benefited by it."

We could perhaps criticise some of Sir Gilbert's positions while attempting to explain the nature and cause of hurricanes; but as it would be wandering from our regular course, we shall only allude to one topic, which is connected with chemical science. "It is ascertained by experiment, (says our author,) that the rays of the sun are not essentially hot, but produce heat by their action on opake bodies, by repeated refraction in passing through pellucid bodies of different specific gravities." We believe that Dr. Hutton first noticed, and Dr. Herschel confirmed the fact, that certain rays of the sun are Calorifers as well as Lucifers; or to use Mr. Brande's words—"it is evident, therefore, that, independent of the illuminating rays, there are others which produce increase of temperature, &c." It is proved, from the place which these calorific rays occupy, after passing through the prism, that they are possessed of less refrangibility than the visible rays of light.* In other respects Sir Gilbert's narrative of this destructive hurricane is blended with much scientific matter, and is indicative of a mind imbued with natural knowledge and strong sense.

The last dissertation in this volume is a short paper on the effects of mechanical compression of the head, as a preventive and cure in certain cases of hydrocephalus, published in our respected cotemporary, the Medical and Physical Journal. We noticed this paper in page 669 of the first volume of this series. Notwithstanding that one or two cases have since been communicated to our author, seeming to corroborate his ideas on this subject, we adhere to the opinion we then delivered, that the principle of external pressure can rarely, if ever, be applicable to the cure of hydrocephalus internus. It is the effect of *pressure* that we have most to dread in all effusions, whether of blood or water within the cavities or investments of the brain, and therefore external pressure, we venture to predict, will be found to aggravate the complaint, *wherever its effects can be unequivocally ascertained.* In Sir Gilbert's own case, leeches and purgatives were used at the same time, and the pressure, *on a child's head*, was so slight as to occasion *no pain or uneasiness.* Under such circumstances it is almost needless to say that no positive indication could be drawn respecting the separate effects of the bandage.

We have now closed the volume, and have to regret that,

---

* See Thomson's Annals, vol. ii. p. 163.

from the great proportion of republished matter, and the nature of the few original articles it contains, we have been able to replenish but a small corner of our journal with its contents. We regret this the more, because, from the course of Nature, we cannot expect that our venerable and highly estimable author will break in much more upon the ease so congenial to old age, by toiling in the literary advancement of his profession. He has already left many honorable and distinguishing marks of an active intellect, and a long and useful life. Let him be contented with these; nor, in future, entangle himself in the labyrinths of medical controversy, where he has little to gain and much to lose. Many of his juvenile opponents are in just the reverse circumstances. They have every thing to gain, (in the way of notoriety) and very little to lose, by the greatest defeat. In plain matters of fact, and in the dignified walks of science, we shall always hail with pleasure the appearance of Sir Gilbert Blane.

